# Practical Notes and Formulæ

**Published:** 1877-10-20

**Authors:** 


### PRACTICAL NOTES and FORMULÆ

Tape Worm.—Dr. Maul kindly furnishes us with the following treatment for tape worm, with which he has succeeded in two cases:

℞. Calomel ..............................

Pulv. ergot, ........aa............. gr. x.

Give every night at bed time.

During the day give every four hours:

Fluid Ex. Nux vomica.............. 3 drops.

Fowlers solution of arsenic........ 5 drops.

To be given in half a teacup full of strong pumpkin seed emulsion. In the one case the worm was discharged on the third day; in the other on the fifth day.

[In Savannah during the prevalence of yellow fever the strange fact was observed that many persons, afflicted with the disease, discharged tape worms whose previous health was good, and who had observed no symptoms of the worm—from which we draw the following practical conclusions:

1st. That persons on the sea coast are more liable to tape worms than in the interior, probably due to fish diet in which they freely indulge.

2nd. The symptoms of tape worm are not so formidable as usually supposed, as many had the worm without knowing it.

3d. That large and repeated doses of *calomel*, or *blue mass*, by its local poisonous effect upon the worm, constitutes a good treatment, as the frequent discharge of tenia in the yellow fever attacks must have been caused by the frequent large doses of calomel which were generally given in these cases.—Ed.]

Bi-Carbonate of Soda.—This is especially useful in all acid conditions of the stomach. In cases of heart burn, or in that heavy, loaded, and sometimes painful state of the stomach, resulting from indigestion, and which usually comes on a short while after eating, nothing gives such speedy and effectual relief as a dose of soda. A half to a teaspoonful may be taken in a little water. We have found soda very useful in that form of irritable and painful micturition caused by acid deposits in the urine. A patient, who had been troubled for fifteen years with what was termed gravel, and "had suffered many things of many physicians," was relieved by the use of a teaspoonful of soda, taken an half hour after each meal. Much of the soda in the shops is crude and impure, and has a strong acrid taste like lye. This should not be used. The milder soda is the best.

Yellow Fever.—Dr. J. J. Knott of this city, furnishes us with his method of treating the above formidable malady. Regarding the affection as dependent upon impressions made upon the excito secretory nerves through the cutaneous system, he uses the spirits of turpentine freely to the entire etxernal surface of the body, and gives ten grain doses of quinine every two hours until the fever abates—at the same time gives small doses of sacharated calomel, with the free use of hot lemonade. He claims that four cases coming from yellow fever districts to this city, were successfully treated by this method, recovery taking place in thirty-six hours. The Doctor has kindly promised to prepare a paper on this subject for our next issue, more fully explaining his theory, and treatment of the disease.

Patent Humbugs.—It is amusing to examine the ingredients which compose our popular patent nostrums, when exposed by the analyst. In *Scientific American* we find the analysis of Dr. Pierce's Golden Medical Discovery as follows:

A $1 bottle holds 220 grains of a brownish colored clear liquid, consisting of 15 grains pure honey, 1 grain extract of poisonous or acrid lettuce, (bot. herba lactucæ virosæ), 2 grains laudanum, 100 grains dilute alcohol (64 per cent.), tasting like fusil oil and wood spirit, with 105 grains of water.

Ayer's Pills consist of pepper, colocynth, gamboge (gutti) and aloes.

Ayer's Hair Vigor, a solution of 0.6 per cent. sugar of lead.

Preserved Summer Squash. — The London *Garden* gives this recipe for preserving summer squash, or "vegetable marrow," as they call it in England: "Mix together four pounds of the fruit, peeled and cut up into small pieces, 3 pounds of white sugar, about a third of an ounce of ground ginger, and the peel of a large lemon cut up small and with the juice squeezed in; boil this mixture for nearly two hours, and it will set firm when cool; it will keep for a year or longer. When properly mixed it makes a preserve of the most delicious kind, and one which would puzzle any one not acquainted with its constituents to tell what it was."—*Boston Jour. Chem.*

REMEDY FOR SORE EYES.—One day an old gentleman of my acquaintance called in and asked me to prepare the following prescription:

Laudanum.... ..................1 ounce.
Sugar of lead.....................3 grains.
Sulphate of quinia.............3 "
Calomel..........................3 "

No directions, no signature, nothing. He wanted it for sore eyes. To make a long story short, I filled the prescription for him, and have for many others since. It is certainly a success in sore eyes. The dir-ctions are to use it with a feather or camel's hair pencil, both externally and internally, *ad libitum.*

DEATH FROM THE WANT OF SALT.—"I am satisfied that I have seen patients die from deprivation of common salt during a protracted illness. It is a common impression that the food for the sick should not be seasoned, and whatever *slop* may be given it is almost innocent of this essential of life. In the milk diet that I recommend in sickness, common salt is used freely, the milk being boiled and given hot. And if the patient cannot take the usual quantitity in his food, I have it given in his drink."—*Scudder.*

DOSES FOR CHILDREN.—Dr. Cowlings' method of calculating doses for children is the following fraction of the adult dose: For a child of one year 2-24, 1-12; two years 3-24, $\frac{1}{8}$; three years, 4-24, $\frac{1}{6}$; five years, 6-24, $\frac{1}{4}$; eleven years, 12-24, $\frac{1}{2}$. It will not do to follow the above rule blindly in every case. In the use of potent remedies, as opium, veratrum, aconite, the above doses may prove too active, while in certain milder articles they are too small.—*Dungleson.*

LOCAL ANÆSTHETIC FOR GUMS.—The following is said to be the mixture in use by traveling Dentists, who boldly advertise the extraction of teeth without pain. It has the effect of benumbing, and to a considerable extent of relieving the pain of extraction. The patient should be cautioned against swallowing any of the fluid.

R. Tinc. aconiti rad...........dr.

Tinc. opii. }

Chloroform. } ....aa.....dr.j.

Alcoholis. } M.

Apply on cotton to the gums until they whiten.

TO TAKE RUST OUT OF STEEL.—Place the article in a bowl containing kerosene oil, or wrap the steel up in a soft cloth well saturated with kerosene; let it remain twenty-four hours, or longer; then scour the rusty spots with brick dust. If badly rusted, use salt wet with hot vinegar; after scouring, rinse every particle of brick dust or salt off with boiling water; dry thoroughly; then polish off with a clean flannel cloth and a little sweet oil.—*Scientific American.*

FORMULA OF INDIAN CHOLAGOGUE.

R. Quiniæ sulphat..............dr.ij.

Pulv. cinchonæ... ...........oz.iv.

Tinct. anguinar...............oz.iv.

Syr. Simplic..................pt.j.

Strychniæ.....................gr.iij.

Acid sulphur. aromat.........dr.ij.

Spts. vini ...... ....... ...oz.ij.

Aq. puræ......................oz.ij.

Ol. gaulther.

Ol. menth. piperit............aa.oz.j.

Misce.

EXCELLENT COUGH MIXTURE.—

R. Morphiæ sulphatis... .......gr. ij.

Acedi sulphuric .............gtt. ij.

M. et adde:

Tinc. serpentariæ............dr j.

Vini antimonii, }

Vini ipecacuanhæ, } ........aa dr. ij.

Tincturæ anisi........... ...dr. j.

Syrupi pruni virginianœ......oz. iv.

M. Tablespoonful every two to three hours.

SUBACUTE RHEUMATIC ARTHRITIS.—The following pill is strongly recommended by Dr. I. S. Davis for rheumatic arthritis:

R. Ferri. sulphat..................dr. j.
Potass. iodid....................scru. iv.
Tragacanth, pulv..............gr. x.
Sach., pulv......................dr. ss.
Syrup...............................q. s.

M. Pills No. XL. Two pills to be taken three times a day.

A Good Liniment.—

R. Tinc. camphor.

Tinc. Opii.

Aqua ammoniæ.............aa oz j.

Oil sasafras.

Oil oregani.

Tinc. capsicum.

M. Chloroform................aa oz ss.

Has a delightful odor, and produces a warm glow and soothing effect in rheumatic or other pains.

Mouth Wash.—The following is recommended as a useful mouth wash for spungy and sore gums; also well adapted to apthae and dyphtheritic affections. After the extraction of teeth it will hasten the healing process and harden the gums.

R. Potassio chlorat................ } aa
Acidi tanici...................... } dr j
Water.........................oz xii
M. Rinse the mouth every three to four hours.

Anti-Hemorrhagic.

R. Pulv. secal. cornut........gr. xxx.
Plumbi acetatis............gr. xx.
Pulv. Opii...................gr. ijss.
Muc. gum. acac............q. s.

Fiat pillula No. 10. Useful in uterine or other hemorrhages, in colliquative diarrhœa, and in night sweats. Dose; one pill every two to four hours.

On Testicles, and the Procreative Power.—Dr. H. A. Spencer, of Erie, Pa., in *Med. and Surg. Reporter*, reports a case of the removal of one testicle, which was followed by no apparent diminution of the procreative power of the individual, he having subsequently begat a number of children.

Hay Fever.—Dr. Evans, in the *Virginia Medical Monthly*, says of hay fever "that there is no agent so powerful fo- good as quinine, given in two grain doses three times a day and continued for several weeks."

Gargle for Diphtheria.—

R. Acidi salicylici............gr. xxv.

Alcoholis.....................oz. ss.

Aquæ distillatæ...........oz. v.

M. Use as a gargle.

Egg Brandy.—Rub the yolks of two eggs with half an ounce of loaf sugar, and add brandy and cinnamon water each four ounces.

